# Optimizing brain stroke detection with a weighted voting ensemble machine learning model

**DOI:** 10.1038/s41598-025-14358-5

**Published:** 2025-08-25

**Authors:** Reeta Samuel, Thanapal Pandi

**Affiliations:** School of Computer Science Engineering and Information Systems, Vellore Institute of Technology, Vellore, 632 014, Tamil Nadu India

**Keywords:** Brain stroke detection, EXtreme gradient boosting (XGBoost), Histogram-based gradient boosting (HGB), Machine learning (ML), Random forest (RF), Weighted voting, Health care, Medical research, Engineering

## Abstract

Brain stroke is a medical trauma that occurs when there is an impairment or decrease in blood circulation to a particular part of the brain, causing adjacent brain cells to die. Stroke diagnosis after an event is an ineffective method; other more labour-intensive and costly procedures exist for stroke diagnosis. This method involves directing a machine learning algorithm to a marked dataset to identify samples and irregularities indicative of stroke occurrence. This study focused on developing an ensemble machine learning model to predict brain stroke. The model combined the predictions of multiple individualistic classifiers, including random forest, eXtreme gradient boosting, and histogram-based gradient boosting, to improve accuracy. The proposed weighted voting-based ensemble (WVE) classifier model achieved an accuracy of 92.31% on a private stroke prediction dataset. The pre-assessment of stroke risk diagnosis, as suggested in this study, enables many people to take preventive actions well in advance, thereby lowering fatal effects. Our proposed method presents a feasible option for the early or initial diagnosis of stroke, as traditional methods, such as computed tomography (CT) scans and magnetic resonance imaging (MRIs), are time-consuming and costly. Future research could explore the use of intelligence-based optimization to enhance classification accuracy and address this limitation.

## Introduction

Stroke continues to be the second most common cause of mortality worldwide and is the primary contributor to long-term neurological impairment^1^. It is a primary contributor to global disability among major muscle-related disorders and holds a prominent position among the top three disorders. Cerebrovascular disease (CVD), characterized by stroke, is a cause of death and morbidity^2^. Moreover, 5 million people worldwide are chronically paralyzed due to stroke, which affects approximately 15 million people annually (Organization, 2015). Brain ischemic stroke, brain hemorrhage, and other severe brain traumas are caused by cerebral vascular disease, which arises from damaged brain blood vessels^3^. There are two categories of stroke: hemorrhagic and ischemic, both of which represent distinct types of occurrences^4^. Ischemic stroke occurs due to blood vessel blockage, whereas hemorrhagic stroke results from blood vessel rupture.

A momentary ischemic attack (TIA) is a form of ischemic stroke characterized by transient blood vessel obstruction^5^. This type of stroke does not cause long-term brain damage and lasts for no more than five hours, in contrast to ischemic stroke^6^. Ischemia or hemorrhage in the brain arteries causes stroke, which is also known as cerebrovascular injury. Stroke can result in many types of physical and cognitive impairments that risk functionality^7^. Brain stroke localization and identification are overly complex tasks that require an accurate affinity for the manner and location of the stroke to implement applicable behavioural interventions^8^.

The prevalent cerebrovascular condition known as ischemic stroke (IS) or cerebral infarction is mostly caused by thrombi obstructing cerebral blood arteries, which results in ischemia and hypoxic necrosis of the brain tissue^9^.

An ischemic stroke occurs when blood clots cause the brain’s blood flow to halt too slowly^10^. After ischemic stroke, patients may also experience stroke bleeding, which can be a dangerous consequence of the disease^11^. After a stroke, the first month of recovery is quick and easy, but the next three to six months are slower^12^.

However, bleeding occurs when a stroke occurs, blood leaks into the surrounding brain tissues, or blood vessels burst owing to their rigidity^13^. The most common causes of hemorrhagic brain stroke are bleeding diseases, aneurysms, arteriovenous disorders, hypertension, and injuries^1^. Hemorrhagic stroke is a severe condition with a high risk of morbidity and mortality (Banjan et al., 2023). AI in radiology, particularly in computer vision and deep learning tasks, is gaining attention owing to advancements such as AlexNet (Liu, X et al.,2021).We also covered several issues in this study, along with potential fixes that need to be explored in further research. The primary outcomes of this study were as follows:

- Proposed a new approach for combining machine learning models using the Weighted Voting-based Ensemble
- (WVE) classifier model.
- Explored a neurological method for classifying brain stroke.
- Employed private data and techniques for diagnosis.
- Then compared our proposed model to various machine learning methods for stroke detection.
- Provides a comprehensive understanding of the patient’s overall health and potential risk factors.
- Determined and examined the obstacles that still need to be solved before assessing the prospects for this field of study.

Motivation for proposed model.

1. Stroke data are complex and diverse, making it difficult for single models to capture all patterns.
2. Basic ensemble methods may not efficiently combine the strengths of these models.
3. Existing methods do not identify risks.
4. Working with private medical data while preserving privacy is a challenge.
5. Single-classifier approaches struggle to capture the complex nature of stroke risk factors.

The Weighted Voting-based Ensemble Classifier model offers several advantages, including achieving 92.31% accuracy, surpassing earlier single-classifier methods. It effectively captures the complex relationships among stroke risk factors, serves as a cost-effective alternative to expensive neuroimaging for diverse healthcare settings, and prioritizes clinical utility with interpretable risk assessments for preventive intervention. Additionally, the model employs an innovative weighted voting mechanism that dynamically adjusts the influence based on the performance.

**Fig. 1:**
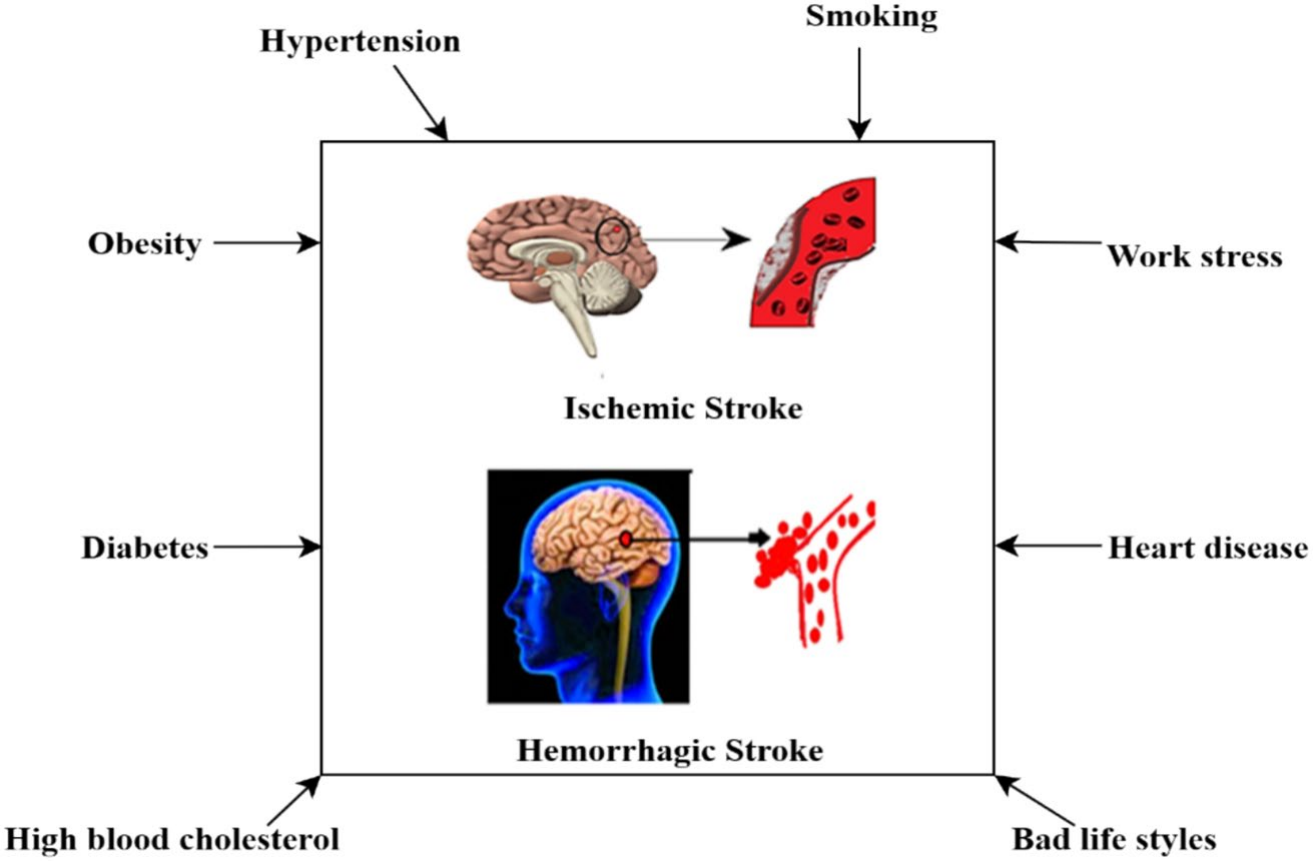
Risk factors for stroke.

Many classification algorithms have been developed in recent decades owing to the vital nature of robotized classification of pictures produced by Magnetic Resonance Imaging (MRI)^14^. It is essential to study and analyze the human mind^15^. MRI’s rich data on delicate tissue living structures have greatly improved our understanding of cerebrum pathology and its remedies^16^. Figure 1 illustrates the risk factors for stroke.

## Related work

Stroke is a significant global health issue, and early and precise detection is crucial for successful treatment. Machine learning has been used to organize brain foci using medical images that resemble CT and MRI scans. One approach involves extracting features from these images, such as texture and shape, and using them to guide machine learning paradigms, such as support vector machines, k-nearest neighbors, or decision trees. Several studies have shown that machine learning methods can effectively classify brain strokes Wang et al.^17^. For instance, a study that used CT scans and support vector machines was able to differentiate between ischemic and hemorrhagic strokes with an accuracy of 85.7%. Another study that used MRI images and convolutional neural networks to group infarctions and edema achieved an accuracy of 94.2%^18^.

Jayachitra and Prasanth developed a new method for segmenting ischemic stroke lesions using fuzzy logic. After segmentation, they extracted features from the segmented regions and used them to train a weighted Gaussian Naive Bayes classifier. Their approach outperformed existing advanced methods in terms of accuracy^19^.

Preprocessing, feature extraction, feature reduction, and brain MRI image classification are the four stages of the conventional classification model. Among all stages of the classification model, preprocessing is the easiest. A noise reduction algorithm was utilized during the preprocessing step to eliminate undesired elements from the images, such as the scalp and skull, and salt-and-pepper noise. The quality of the images was enhanced by removing noise^20^.

Ultrasound (US) is one of the most used medical diagnostic methods and is an essential tool for medical imaging. It is crucial for both qualitative and quantitative disease classification and, medical assessments. The advantages of ultrasound imaging over other medical imaging technologies include its low cost, ease of use, lack of ionizing radioactivity, great understanding, and simultaneous imaging. Nonetheless, US imaging has certain issues compared with CT, MRI, and X-ray imaging. For example, increased noise and artifacts and reduced tissue contrast lead to boundary challenges. (Wu, L et al., 2017).

Prior research has used machine learning techniques to forecast motor and working healing during the critical and subacute cycles of stroke^21^. Machine learning models have been used to predict stroke patients’ recovery of motor or intellectual abilities^22^. Researchers have developed various screening techniques to improve the effectiveness of stroke screening, which can be broadly categorized into four categories. By implementing revisions to the detection evaluation form for the stroke population, the screening efficiency was enhanced^23^.

Hung et al. extended a deep convolutional network representation using Taiwan’s stroke diagnosis record (EMC) and contrasted its outcomes with those of conventional machine learning techniques. The AUC of 0.92 represented the highest accuracy between the two divisions (stroke or no stroke). Although the trial range in this study was rather large and could identify strokes with some precision, the overall result was not favourable^24^.

S. J. Hegland et al. developed a predictive model for acute strokes using CNN and brain MRI data. The best result for the deep convolutional network for ischemic stroke prediction was an AUC of 0.88 ± 0.12^25^. Immediately after stroke, brain impairment results from blood flow disruption^26^.

This study aimed to enhance the effectiveness of brain–computer interface (BCI) technology for stroke recovery. The researchers created a new method for recognizing a patient’s intention to move, utilizing a technique called"time series shapelets."This innovative approach demonstrated superior performance compared to existing methods in both offline analysis and simulated real-time scenarios^27^.

Z. Gong et al., developed a new approach to microwave medical sensing (MMS) is presented, enabling rapid and accurate classification and localization of strokes. By dividing the examination area into sections and utilizing decision tree learning, this method efficiently identifies the stroke characteristics. Compared with traditional methods, it achieves a 14.1% and 19.2% improvement in classification rates, reduces localization time by 21.1%, and attains a localization accuracy of over 0.90. This innovative space-division-based technique is particularly suitable for wearable devices and offers a promising solution for localizing brain strokes without the need for imaging^28^.

This research explored the application of Huygens’ principle (HP) imaging for stroke detection in the brain. Although the intricate structure of the brain poses challenges, recent advancements in artificial intelligence and deep learning have enhanced the accuracy of stroke detection and classification. Through simulations using the Finite Difference Time Domain (FDTD) method, we revealed that combining the magnitude and phase information from HP imaging improves stroke detection and classification accuracy. The proposed approach was validated using real-world data from two patients^29^. Table 1 presents a comparison of the existing models.

**Table 1:** Comparison of existing model in literature.

Key technique	Model	Research performance	Limitation	References
Machine learning and deep learning	CNN, LSTM, KNN, XGB, and majority voting ensemble	Proposed model obtained the highest classification performance based on all evaluation metrics on all datasets	Potential limitations in generalizability to other populations or datasets, need for further validation, May require significant computational resources	^30^
Deep learning	CNN-GRU, SMOTE Method	Higher classification accuracy compared to other existing models	Potential limitations in generalizability to other datasets or environments	^31^
Ensemble learning, data mining techniques	Weighted ensemble model using genetic algorithm	Improved performance compared to individual classifiers	May require significant computational resources	^32^
Remote monitoring	Web application for remote monitoring and management, Real-time monitoring and alerts	Effective in monitoring and managing high-risk pregnancies	Limited to healthcare professionals, not designed for patient use	^32^
Ensemble-based deep learning model	CNN, LSTM, XGBoost, KNN	Outperformed existing models, demonstrating superiority in cardiovascular disease prediction	Lack of interpretability of the model’s predictions due to the complexity of the ensemble architecture	^33^
Semantic relatedness and similarity measures	natural language, machine learning algorithms	Using students’ answers as feedback considerably improved the accuracy and performance of these measures	The dataset used is relatively small	^34^
Machine learning	Neural networks, SVM, KNN	remarkable accuracy and minimal loss	Limited to a single dataset, potential variation with other datasets	^35^
Machine learning	Nomogram prediction model	Successfully identified several parameters associated with stroke risk, demonstrated superior predictive accuracy	Potential limitations in generalizability to other populations, need for further validation	^36^
Machine learning (ML)	Random forest (RF), KNN, DT, AdaBoost, XGBoost, SVM, ANN	RF achieved highest performance	Potential limitations in generalizability to other populations or datasets, need for further validation, May require significant computational resources	^37^
Ensemble Machine Learning	Soft Voting Classifier (Random Forest, Extremely Randomized Trees, Histogram-Based Gradient Boosting)	Achieved an accuracy of 96.88%, improved accuracy and robustness compared to single classifiers	Potential limitations in handling complex interactions between features, need for further optimization	^18^
Face Detection using Yolo v8	Stroke monitoring strategy	Achieved high accuracy of 98.43%	Limited availability of stroke patient data	^38^
Modified Vision Transformer (ViT) integrated approach	End to end ViT Architecture, CNN	87.51% classification accuracy for brain CT scan slices	Improvement needed for stroke diagnosis	^43^
A deep-learning-based Microwave-induced thermo acoustic tomography MITAT (DL-MITAT) Technique	A residual attention U-Net (ResAttU-Net)	effectively eliminated image artifacts and accurately restored hemorrhage spots as small as 3 mm	No performance metrics for increased accuracy; training sets are constructed only using the simulation approach	^44^
AutoML	A combination of AutoML, Vision Transformers (ViT), and CNN	The model achieved 87% accuracy for single-slice level predictions and 92% accuracy for patient-wise predictions	Small sample size, complexity of the integrated architecture	^45^

## Proposed model

We propose a Weighted Voting-based ensemble classifier and compare it with seven common machine learning algorithms: Logistic Regression (LR), Support Vector Machines (SVM), Decision Tree (DT), Random Forest (RF), gradient tree boosting (GB), K-Nearest Neighbor (KNN), and Naive Bayes (NB) and employed a voting classifier in this study.

**Fig. 2:**
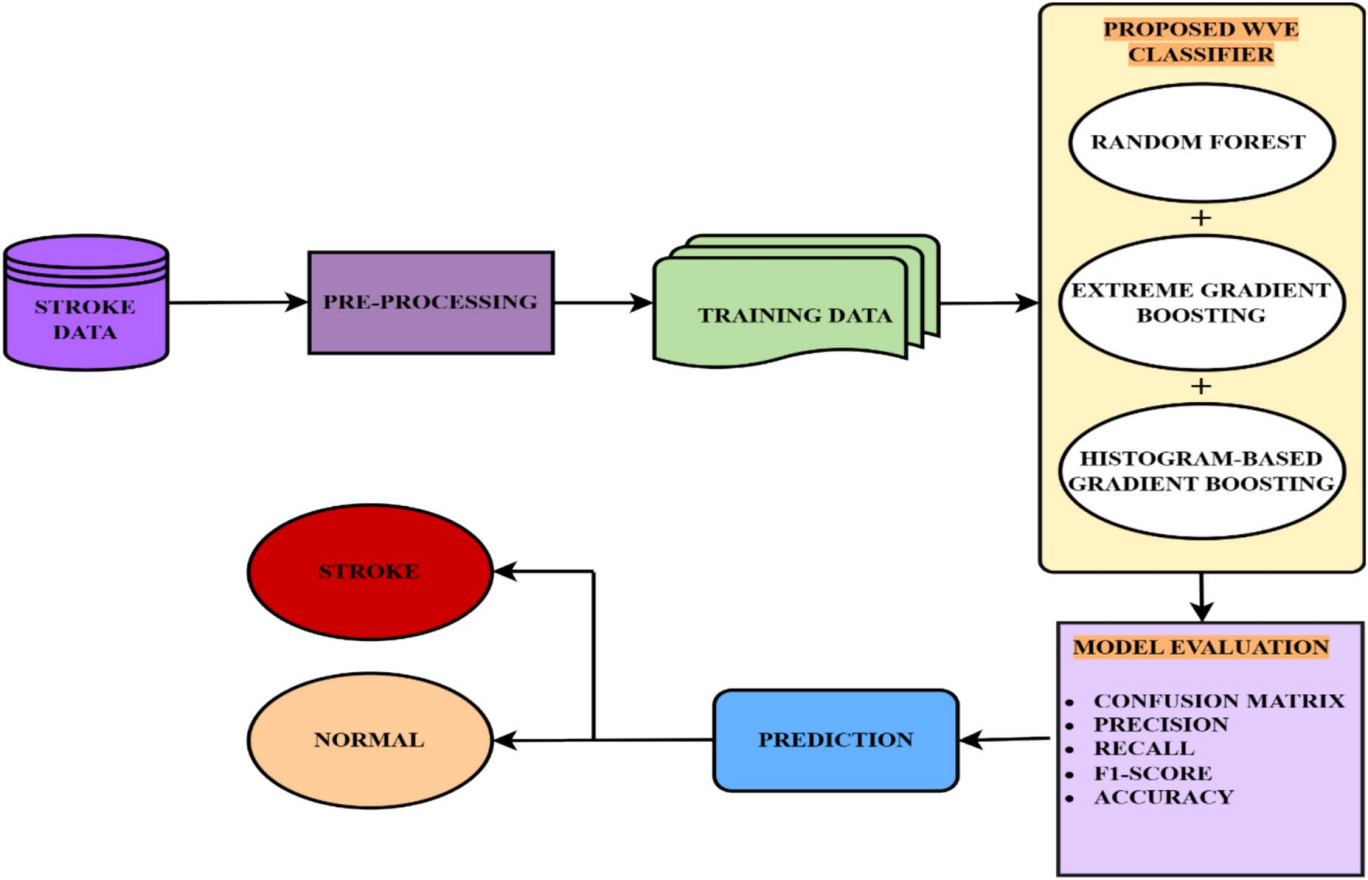
Proposed weighted voting-based ensemble classifier model.

A Voting classifier combines multiple models to make predictions by choosing the class with the maximum possibility. It is often used to forecast outcomes, such as voting results. Because the weighted voting ensemble considers each base classifier’s optimism in its prediction, compared to hard voting, which sums the number of times each model has been recognized by crucial classifiers, it is usually considered more accurate and dependable. It is an easy-to-use technique that can be used to improve the performance of a machine-learning model in both classification and regression scenarios. Figure 2 illustrates the proposed model.

Three basic classifiers are utilized in the proposed WVE classifier: Random Forest, eXtreme gradient boosting, and histogram-based gradient boosting. The nomenclature is presented in Table 2. The following is an overview of each base classifiers.

**Table 2:** Summary of notations.

Notation	Description
$\widehat{{y}_{i}}$	Final predicted output for the i^th^ input sample
${x}_{i}$	The i^th^ input data sample
${T}_{n}{(x}_{i})$	Predicted class label by the n^th^ model or client for input ${x}_{i}$
	Summation overall N models or clients
$\frac{1}{N}$	Averaging factor to compute the mean prediction from all contributors
$N$	Total number of models or clients
$mode()$	Statistical mode function that returns the most frequent class label
$L\left(\theta \right)$	Total loss function with parameters $\left(\theta \right)$
$n$	Total number of data samples
${\text{l}(\text{u}}_{i},{\widehat{u}}_{i})$	Loss between ground truth ${\text{u}}_{i}$ and predicted output ${\widehat{u}}_{i}$
	Summation overall n training samples
	Summation over all K model components
$\Omega {(f}_{k})$	Regularization term for the kth model component
${(f}_{k})$	Model parameters of the k^th^ component
$\left(\theta \right)$	Overall set of model parameters
${\gamma }^{T}$	Bias or constant term related to iteration T
$\lambda$	Regularization coefficient
$T$	Total number of training iterations or time steps
${w}_{j}^{2}$	Model weight parameter at step j
$\sum_{j=1}^{T}{w}_{j}^{2}$	Sum of squared weights
$\frac{1}{2}\lambda \sum_{j=1}^{T}{w}_{j}^{2}$	L2 regularization term
${f}_{k} {(x}_{i})$	Output of the k^th^ model when applied to input ${x}_{i}$
$K$	Total number of models contributing to the aggregation
	Summation over all K models
${\widehat{Y}}_{k}$	An estimated value at index k
	Summation from j = 0 to j = n, so summing up n + 1 terms
${Y}_{k}^{\left(j\right)}$	Classifier
${w}_{j}$	Weight assigned to j^th^ classifier

### Random forest

Random Forest (RF), a supervised learning technique, is used for regression and classification tasks. It consists of decision trees (also called “forest”), bagging, feature randomness, and voting. It can handle high-dimensional data, prevent overfitting, manage missing values, and is interpretable.

**Fig. 3:**
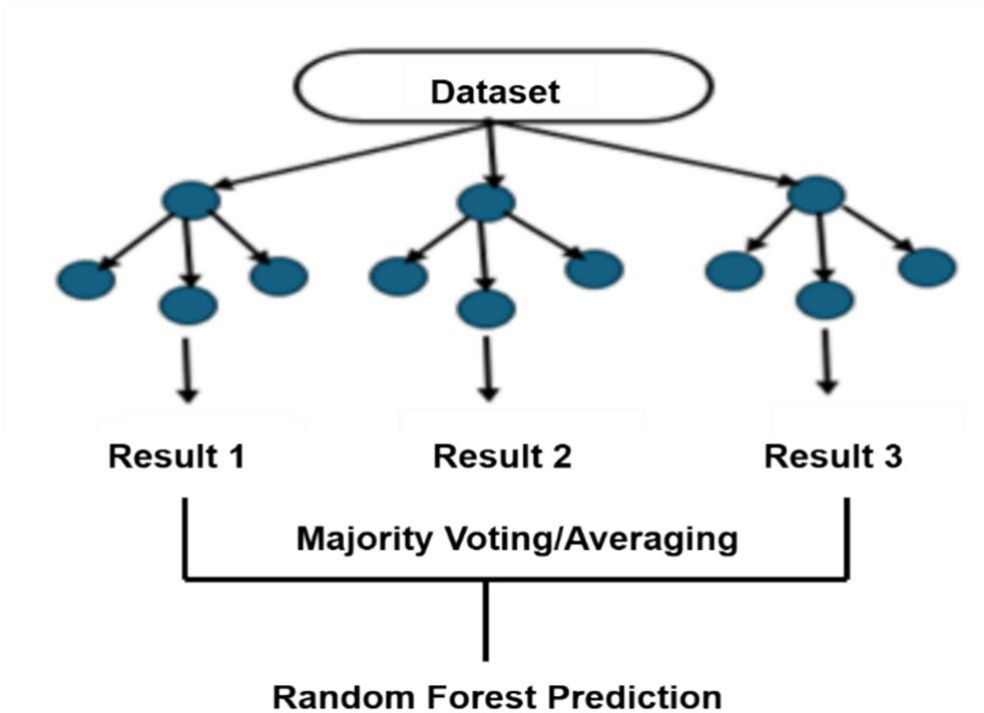
Random forest classifier.

The hyperparameters control the ensemble size, depth, features, and sample split. Common applications include image classification and regression. At the Random Forest level, the average feature importance across all trees was the final measure of significance. The importance value of each characteristic of each tree was added together and divided by the total number of trees. For regression, a random forest prediction was used.

1 $$\widehat{{y}_{i}}=\frac{1}{N} \sum_{n=1}^{N}{T}_{n}{(x}_{i})$$

2 $$\widehat{{{ }y_{i} }} = { }mode\left( {T_{1} \left( {x_{i} } \right),T_{2} (x_{i} } \right),{ } \ldots .{ },{ }T_{N} (x_{i} ))$$

where N is the sum of trees and ${T}_{n}{(x}_{i})$ is the calculation from the n-th tree for input ${(x}_{i})$. For classification, the final prediction is the mode (majority vote) of the class prediction from all trees: where ${T}_{N}{(x}_{i})$ is the prediction from the n-th tree for input ${(x}_{i})$. Random Forest builds various individual decision trees and associates them through averaging (regression) or voting (classification). However, Random Forest is not always the best choice, therefore, it is essential to experiment and compare its performance with that of other algorithms. Regarded as one of the most potent and resilient algorithms available, Random Forest is simple to operate and capable of processing a multitude of characteristics and categorical variables. In addition, it is less likely to overfit than a single decision tree. Figure 3 shows the Random Forest graphic depiction.

### XGBOOST, or extreme gradient boosting

A very effective and adaptable gradient boosting framework is called Extreme Gradient Boosting (XGBoost). It is intended to outperform conventional gradient boosting techniques in terms of scalability, regularization, and speed. Owing to its exceptional performance, XGBoost has become popular in both real-world applications and machine learning competitions.

Key characteristics of regularization with XGBoost: XGBoost uses L1 and L2 regularization to reduce overfitting and improve generalization. System Optimization: It suited for huge datasets because it is optimized for parallel and distributed computing. Flexibility: XGBoost can process both arithmetic and categorical attributes across a wide range of data formats. Efficiency: It is appropriate for large-scale challenges because of its computational efficiency. XGBoost contains built-in techniques for handling missing data. Customizable Loss Functions: Custom loss functions can be built to adapt the algorithm to challenges. The regularized objective function with two components is reduced by XGBoost as follows:

3 $$L\left(\theta \right)=\sum_{i=1}^{n}l{(\text{u}}_{i},{\widehat{u}}_{i})+\sum_{k=1}^{k}\Omega {(f}_{k})$$

where, $l{(\text{u}}_{i},{\widehat{u}}_{i})$ is the loss function that measures how well the model fits the data, represents the total number of data points, ${\text{u}}_{i}$ is the actual observed value for the i^th^ data point, and ${\widehat{u}}_{i}$ is the predicted value for the i^th^ data point. $\Omega {(f}_{k})$ is the regularization term for the complexity of the k^th^ tree, which is usually defined as

4 $${\Omega {(f}_{k})=\gamma }^{T}+\frac{1}{2}\lambda \sum_{j=1}^{T}{w}_{j}^{2}$$

where:

- $\gamma$ is a regularization parameter that controls the number of leaves in the tree.
- $T$ represents the number of terminal nodes in the tree.
- ${w}_{j}$ is the weight of each leaf.
- $\lambda$ is a regularization parameter for leaf weights. The predicted value for an input x is calculated by adding the outputs of all the trees:

where ${f}_{k}$ ${(x}_{i})$ is the prediction output of the k^th^ tree, and K is the total number of trees.

5 $${\widehat{y}}_{i}= \sum_{k=1}^{K}{f}_{k} {(x}_{i})$$

### Histogram based gradient boosting

**Fig. 4:**
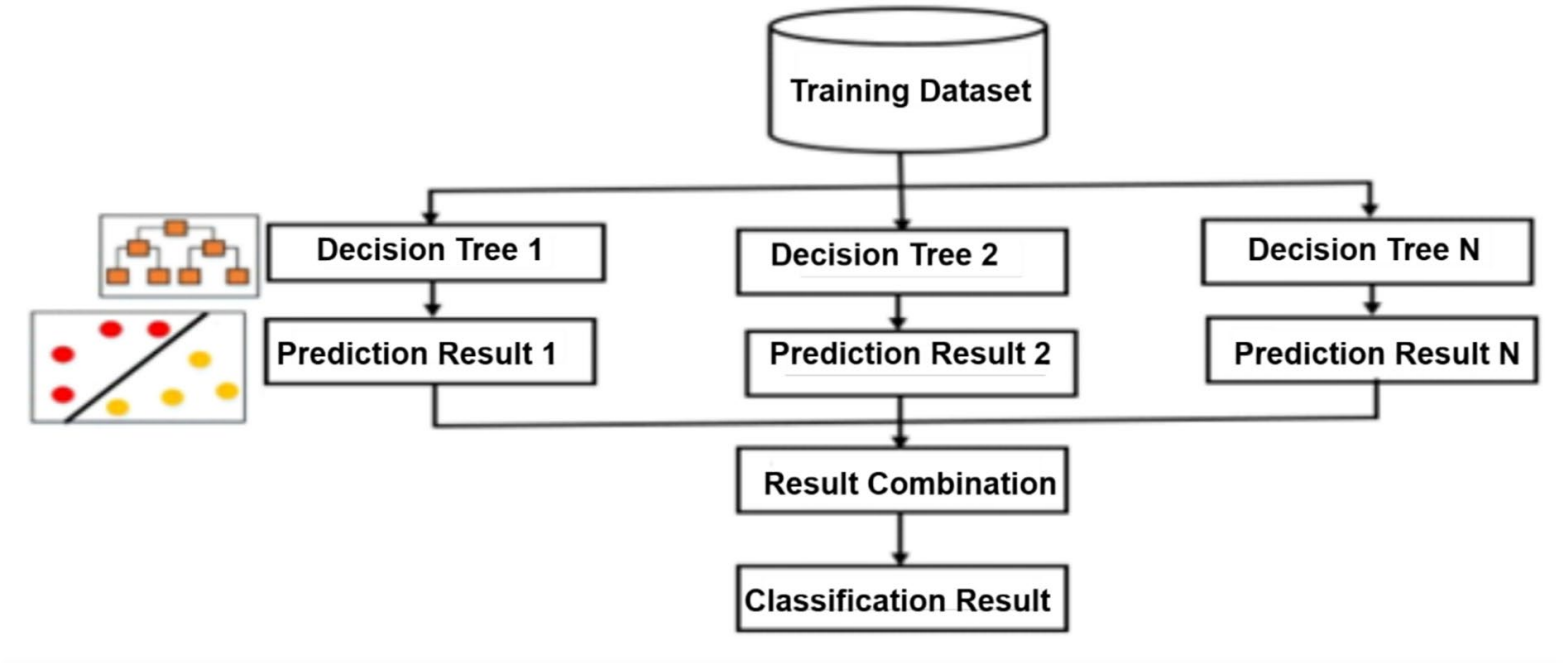
Histogram-based gradient boosting structure.

Histogram-Based Gradient Boosting, or HBGB for simple terms, remains the machine learning equal to the Gradient Boosting algorithm. A sophisticated ensemble technique called gradient boosting constructs a model by integrating the predictions from numerous ineffective models, each of which is instructed to fix the mistakes of the previous models. Rather than employing a single decision tree, as in the past, HBGB uses histograms to approximate the underlying data distribution. It creates a histogram for each characteristic and divides the data into distinct bins based on the histogram of each feature. Subsequently, each bin was assigned a decision tree model. The HBGB aims to reduce an objective function that consists of a loss term and regularization term. The most used loss function for regression is the mean squared error (MSE). For classification, the log loss is declared as Eq. 7.

6 $$l{(\text{u}}_{i},{\widehat{u}}_{i})={{(\text{u}}_{i},{\widehat{u}}_{i})}^{2}$$

7 $$l{(\text{u}}_{i},{\widehat{u}}_{i})=-{u}_{i}\text{log}\left(\widehat{{u}_{i}}\right)-\left(1-{u}_{i}\right)\text{log}\left(1-{\widehat{u}}_{i}\right)$$

where $l$ is the loss function that quantifies how well the model fits the data, n is the total number of data points, ${u}_{i}$ is the true value for the i^th^ data point, and ${\widehat{u}}_{i}$ is the predicted value for the i^th^ data point. This can improve performance by enabling the procedure to more closely approach the data’s essential spreading Fig. 4 shows the structure of histogram based gradient boosting. Because HBGB can conduct these patterns better than the usual gradient boosting, it is especially well-suited for datasets with many features, severely skewed data, or data with outliers. It can also be parallelized to expedite training, and is reasonably quick and simple to use.

## Findings and discussion

This section discusses the results of the proposed WVE classifier and comparative analysis. A private dataset comprising stroke patient records was collected from text files (Excel file) at KC Multi-Specialty Hospital in Chennai, India. We confirm that all methods and experiments conducted were purely computational and did not involve any human subjects directly. The patient’s details were highly confidential.

- We confirm that all methods were carried out in accordance with relevant guidelines and regulations.
- We confirm that all experimental protocols were approved by the KC Multispecialty Hospital, Chennai, India.
- We confirm that informed consent was obtained from all subjects and/or their legal guardian(s).

This dataset was used to compare various machine learning algorithms with the proposed model. This study used a private dataset comprising 280 records. After implementing quality assurance measures, 261 high-quality records were selected for analysis. Among these, 87 records were labeled as stroke cases (assigned a value of 1), and the remaining 174 were classified as normal (assigned a value of 0). To evaluate the model performance, the dataset was divided into a train set (80%) and a test set (20%). Extensive data preprocessing was performed to ensure consistency and accuracy of the analysis. This dataset provides valuable and distinct insights. To avoid the model deviating from the intended training data, data preprocessing is necessary before model construction to eliminate superfluous noise and outliers from the dataset. The dataset contains 11 characteristics. Table 3 shows the data sample format. The performance of these algorithms was evaluated using standard metrics, including accuracy, precision, recall, and F1-score.

**Table 3:** Sample data format.

Gender	Age	Hypertension	Heart_ disease	Ever_ married	Job type	Residence type	Avg_ Glucose level	BMI	Smoking status	Stroke
Male	57	0	1	No	Govt	Urban	217.08	33.80841	Unknown	1
Male	58	0	0	Yes	Private	Rural	189.84	31.37853	Unknown	1
Female	58	0	0	Yes	Private	Urban	71.2	30.00388	Unknown	1
Male	58	0	0	Yes	Private	Urban	82.3	30.19957	smokes	1
Female	59	0	0	Yes	Private	Rural	211.78	33.48457	formerly smoked	1
Male	79	0	1	Yes	Private	Urban	57.08	22	formerly smoked	0
Female	37	0	0	Yes	Private	Rural	162.96	39.4	never smoked	0
Female	37	0	0	Yes	Private	Rural	73.5	26.1	formerly smoked	0

**Fig. 5:**
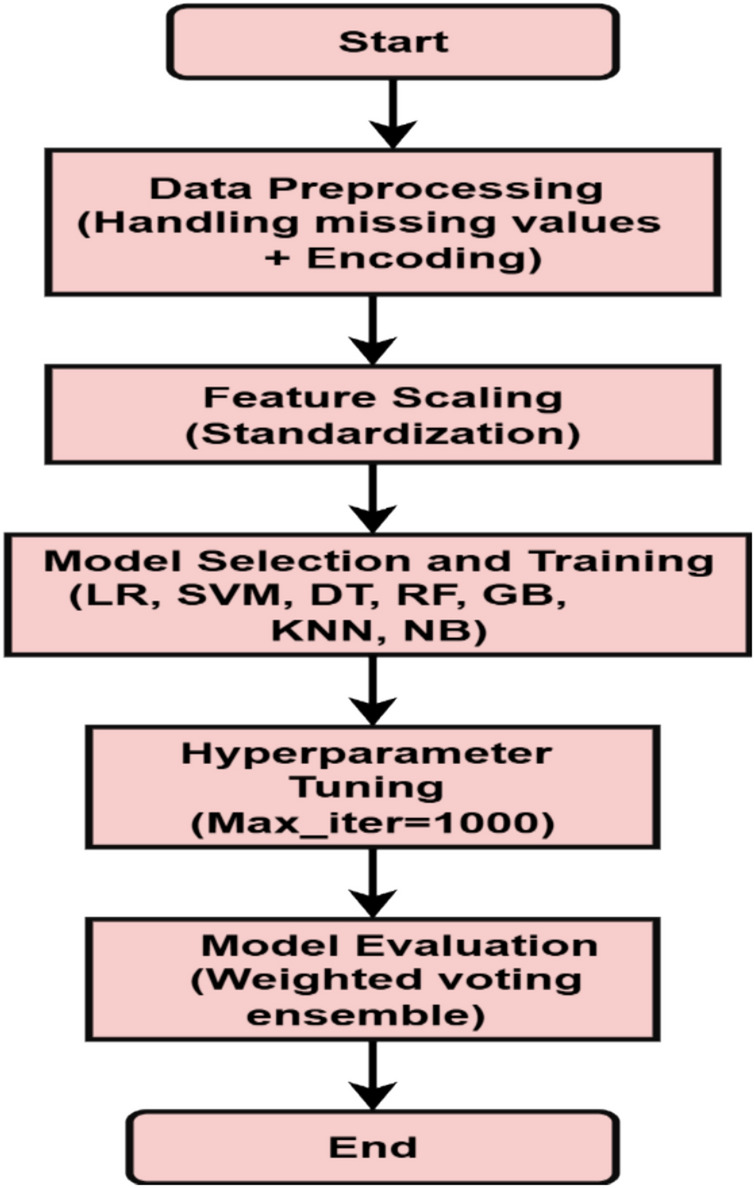
Overview of the implementation process.

The dataset contains 11 features for each sample and a target variable. The target variable was binary, with 1 representing stroke and 0 representing no stroke. A brief overview of these features is provided in Table 4. Figure 5 presents an overview of the implementation process.

**Table 4:** Feature description for the dataset.

Feature	Description
Gender	Male, female, others
Age	Age of the patient
Hypertension	0 = no hypertension, 1 = has hypertension
Heart disease	0 = no heart disease, 1 = has heart disease
Ever married	Patient’s marital status
Job type	Patient’s work type
Residence_type	Patient’s residence type
Avg_Glucose level	The average glucose level in the blood
BMI	body mass index
Smoking status	Smoking status: formerly smoking/never smoked/smoked
Stroke (Target)	0 (zero) = no stroke, 1(one) = has stroke

The dataset was pre-processed to handle missing values and normalize the numerical features. We maintained the original class distribution without applying any resampling techniques to preserve the real-world imbalance in stroke occurrence. Our dataset consisted of clinical and demographic data, including variables such as age, gender, hypertension, and heart disease. The data are tabular and are used to predict stroke risk or diagnose stroke.

o Total samples: 261 selected from 280 records
o Feature dimensions: 11 features after one-hot encoding
o Class distribution: 87 positive (stroke) and 174 negative stroke cases
o Data source: Clinical records were collected from hospital data and stored as CSV file.

We used the Stroke Prediction Dataset, which has the following features:

o Demographic information (age, gender)
o Medical history (hypertension, heart disease)
o Lifestyle factors (smoking status, BMI)
o Socioeconomic indicators (work type, residence type, average glucose level)
o Laboratory results (average glucose level, etc.)

**Fig. 6:**
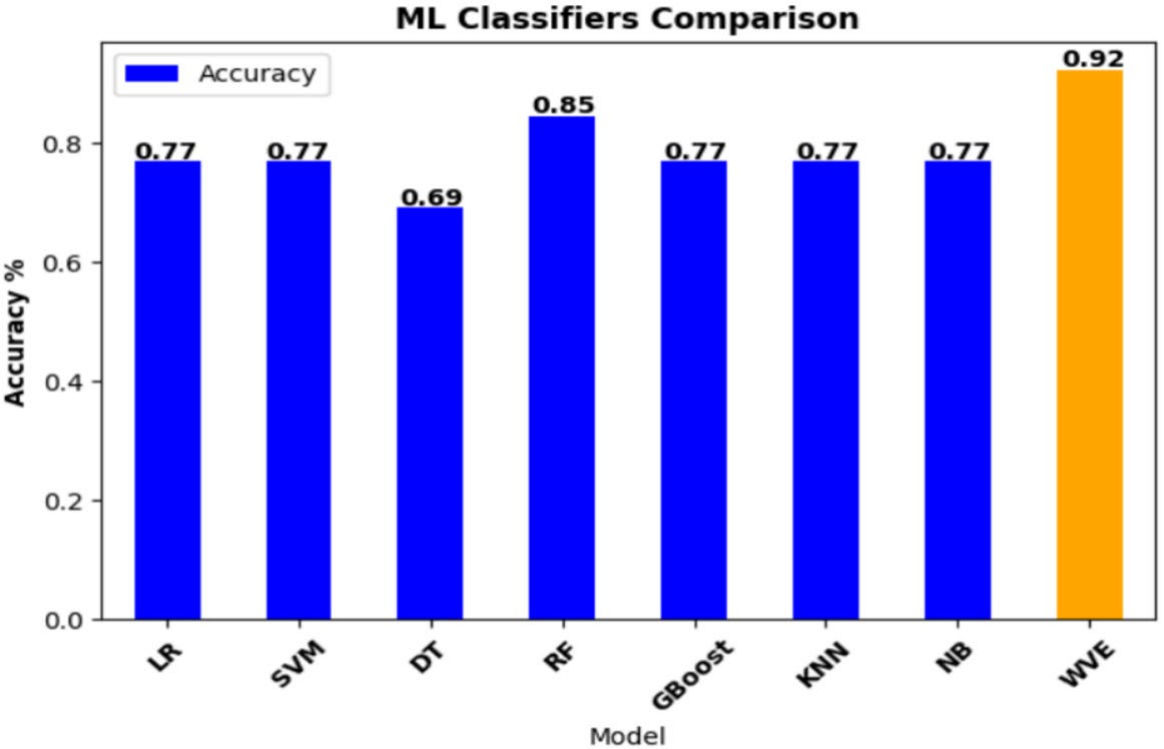
Comparison of the proposed construction with alternative models.

**Fig. 7:**
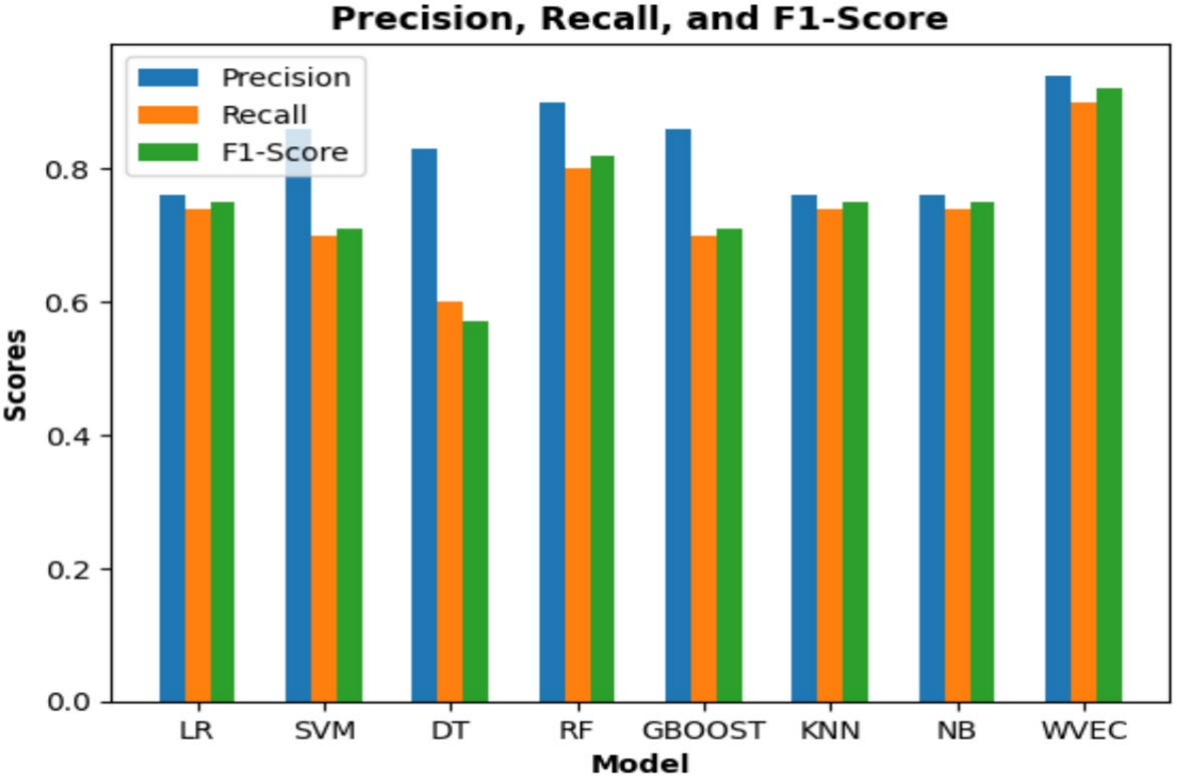
An analysis of the proposed model’s precision, recall, and F1-score.

Stroke affects older individuals, with most patients aged between 60 and 80 years. While men experience strokes earlier, typically in their mid-50 s to 80 s, women are commonly affected between their late 40 s and 80 s of age. The data revealed that a substantial proportion of patients, particularly men, were overweight or obese. Some patients have extremely high BMIs. Interestingly, although heart disease is not prevalent among patients with stroke, high blood pressure is not a common risk factor. Additionally, a larger number of patients maintained normal blood sugar levels. Table 5 presents the performance of different machine learning methods with the proposed model in predicting brain strokes. Implemented a machine learning (ML) technique was implemented using a WVE classifier in the proposed system. The proposed approach is tested using several machine learning techniques, with Logistic Regression, SVM, Decision Tree, Random Forest, Gradient Tree Boosting, KNN, and Naive Bayes. Based on their accuracy scores, the best individual classifiers were used in the ensemble voting classifier. Figure 6 shows a comparative evaluation of the suggested framework with other models. The general F1 score obtained in this study was 92%. This model was fine-tuned to the highest possible degree after several iterations. The model attained an accuracy of 92.31%. The proposed model achieved a maximum accuracy of 92%, recall of 90%, F1-score of 92%, and precision of 94%. Figure 7 shows the precision, recall, and F1-score.

**Table 5:** Evaluation of performance metrics of the proposed model.

Model	Precision (%)	Recall (%)	F1-Score (%)	Accuracy (%)
Logistic regression	76	74	75	77
Support vector machines	86	70	71	77
Decision tree	83	60	57	69
Random forest	90	80	82	85
Gradient tree boosting	86	70	71	77
K-nearest neighbor	76	74	75	76
Naive bayes	76	74	75	76
**Proposed model**	**94**	**90**	**92**	**92**

A confusion matrix is a pictorial tool commonly used in machine learning to evaluate the execution of classification models. It presents a tabular representation of the expected and real class labels, providing a comprehensive evaluation of the model’s accuracy. The rows of the matrix represent the true class labels, and the columns represent the predicted class labels of the model. The slanted elements indicate correct classifications, whereas the off-diagonal elements denote the misclassifications.

Many methods are currently employed to identify stroke disease, but the most underutilized method is preliminary stroke risk assessment based on critical factors, including age, blood glucose level, hypertension, and body mass index. A comprehensive analysis of the predicted data for the new patients was conducted. BMI was classified as normal (18.5–24.9), overweight (25–29.9), obese (30–34.9), or extremely obese (> 34.9). Additionally, glucose levels were classified as normal (170–200), elevated (190–230), or high (220–300). These findings, combined with stroke risk levels, provide a comprehensive understanding of a patient’s overall health and potential risk factors for stroke.

**Fig. 8:**
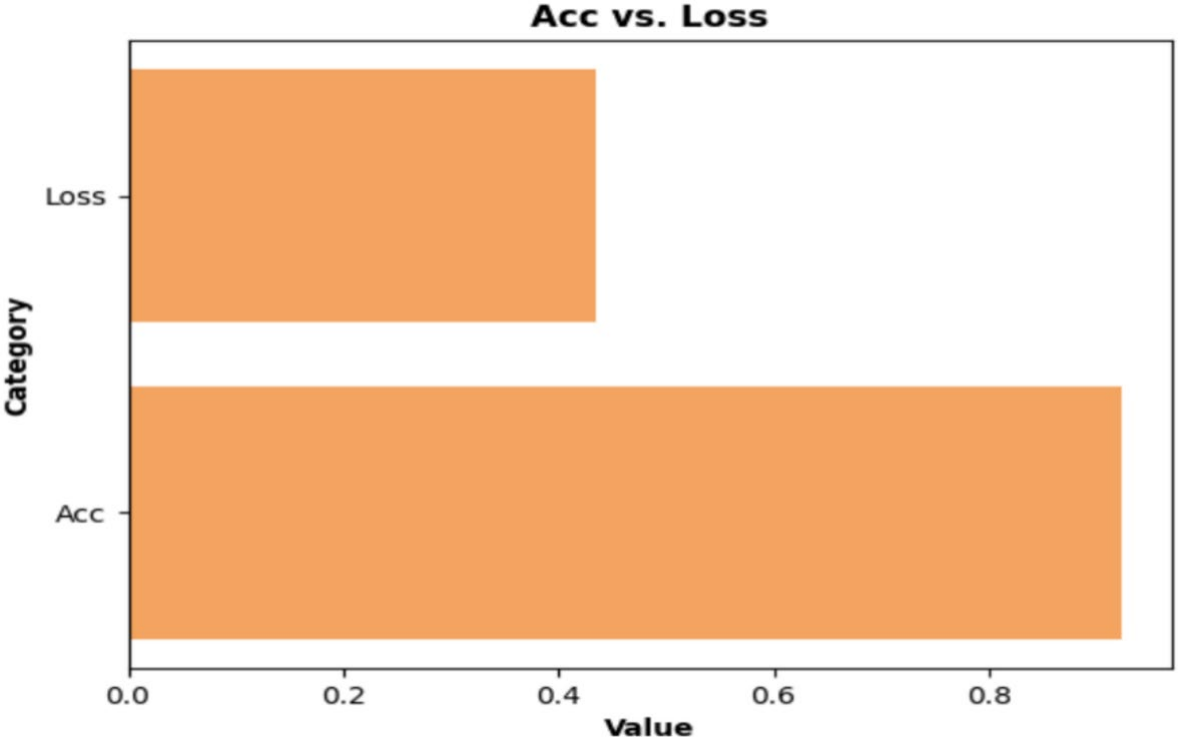
Accuracy and log loss interpretation.

In addition, we determined the accuracy of the proposed structure to be 0.9231, and its log loss to be 0.4351. These values were calculated to facilitate comparisons with the existing models, as shown in Fig. 8. The models used in our ensemble Voting Classifier (RandomForestClassifier, XGBClassifier, and GradientBoostingClassifier) are traditional machine learning algorithms and do not rely on epoch-based training. These ensemble methods do not use epochs, as neural networks. Instead, they built decision trees based on bootstrapped samples. The accuracy (0.9231) and log loss (0.4351) metrics shown in Fig. 8 were not achieved through epoch-based training.

The final performance metrics were obtained from a single training and evaluation cycle on the test data after fitting the models. The relationship between accuracy and log loss is a result of the probability calibration from the soft voting method, where class probabilities from all base estimators are averaged. The reported metrics represent the ensemble’s performance on unseen test data, suggesting that the model generalizes well with minimal overfitting.

**Fig. 9:**
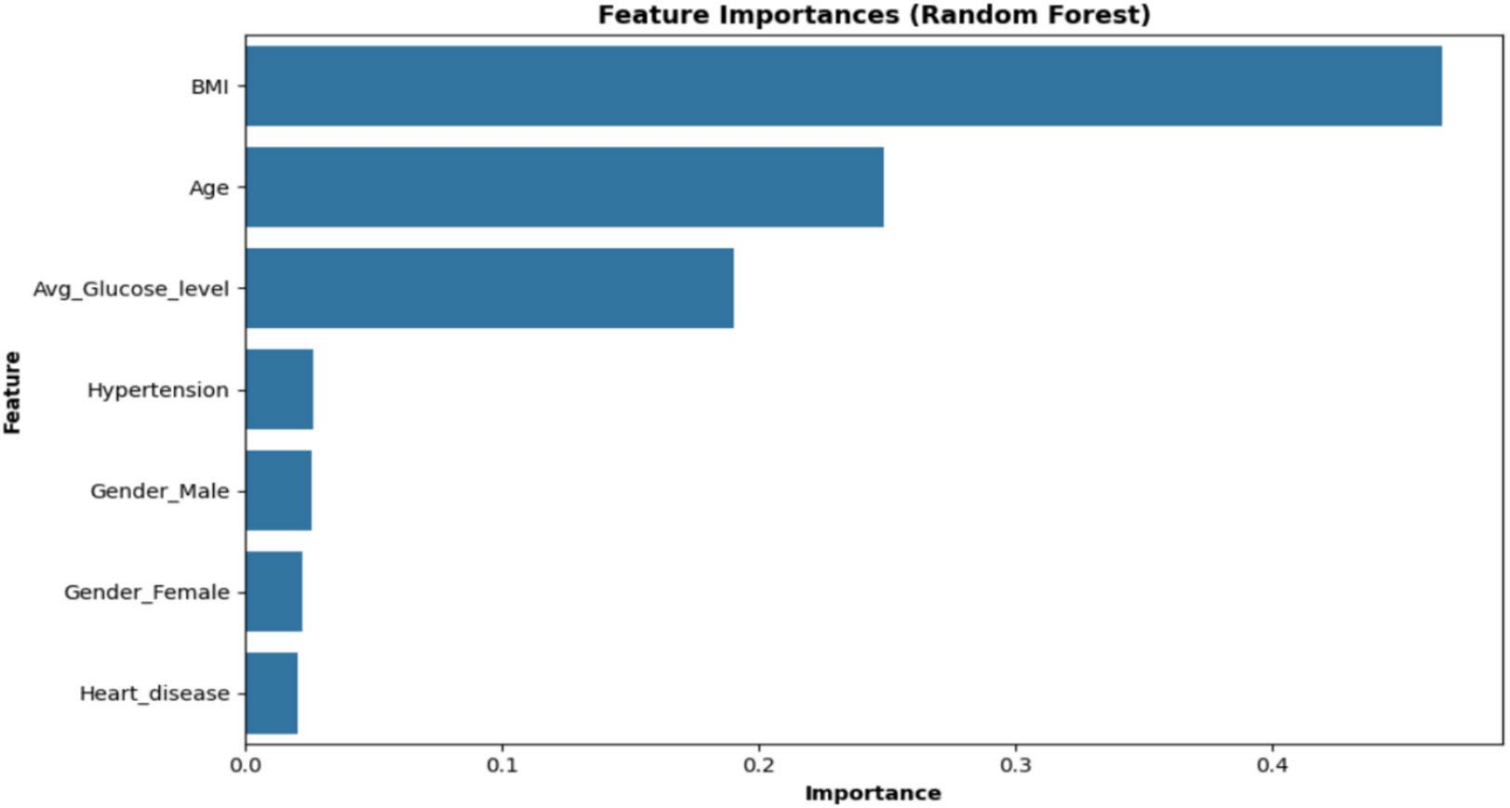
Feature importance ranking.

This mathematical equation describes the process of weighted voting-based ensemble classification, and the final predicted class is determined by choosing the class with the highest average probability among all models. Figure 9 illustrates the importance of the Random Forest model, which shows the relative implication of each variable in predicting outcomes. By analyzing the frequency with which features are used to split the data within the decision trees, we can identify the most influential factors. This information is valuable for selecting relevant features, understanding the decision-making process of the model, and exploring the underlying relationships between variables. However, potential limitations, such as correlation and non-linearity, should be considered.

A voting classifier equation is proposed using a weighted average approach for every prediction model, defined as follows in Eq. 8, where the weight must be a specific value. ${w}_{j}$ represents the weight assigned to each classifier. Where *m* represents the individual classifiers. ${Y}_{k}^{\left(j\right)}$ are the classifiers. The approximate probabilities p can be calculated for the models as follows in Eq. 9, where ${w}_{j}$ = weight assigned to j^th^ classifier. The final prediction $P\left(x\right)$ for input $x$ is determined by the weighted sum of the classifier predictions, as expressed in Eq. 10.

8 $${\widehat{Y}}_{k }= {\sum }_{j=0}^{n}{w}_{j }{Y}_{k}^{\left(j\right)} , where {w}_{j}>0$$

9 $$\widehat{y }=\text{argmax}{\sum }_{j=0}^{m}{w}_{j} {p}_{ij}$$

10 $$P\left(x\right)=\left\{\begin{array}{c}1, if{\sum }_{i=1}^{N}{w}_{i}.{C}_{i}(x) \ge T \\ 0, otherwise \end{array}\right.$$

where:

- $T$ is the decision-making threshold.
- ${w}_{i}$ is the weight of the i^th^ classifier, based on its performance.
- $N$ is the total number of classifiers in an ensemble.
- ${C}_{i}(x)$ be the prediction of the i^th^ classifier for input $x$, where $i\in \left\{\text{1,2},\dots ..,N\right\}.$
- ${w}_{i}$ be the weight allocated to the i^th^ classifier, where ${w}_{i}\ge 0 and {\sum }_{i=1}^{N}{w}_{i}=1.$

For the Binary Classification problem, which is stroke and no stroke, where 0 indicates no stroke detected and 1 indicates stroke detected.

$${C}_{i}\left(x\right)\in \left\{\text{0,1}\right\}$$

Input: The function takes a data point, a list of trained models, and a list of corresponding weights as input.

Weighted Probabilities: It iterates through the models, obtains the predicted probabilities for each class, and multiplies them by the corresponding weights. The weighted probabilities were accumulated for each class.

Prediction: The function returns the class with the highest accumulated weighted probability as the predicted class.

**Figure:**
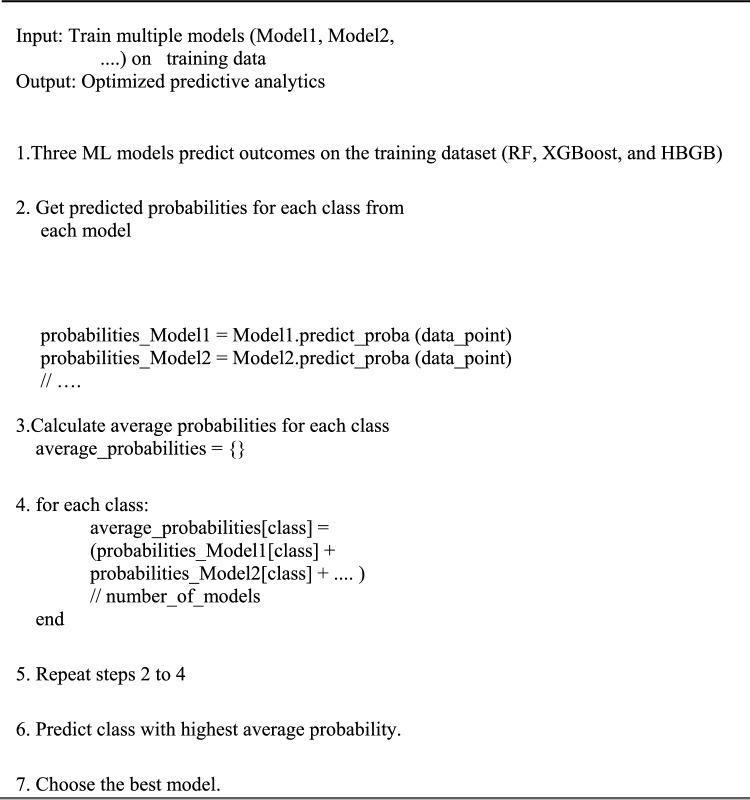
Algorithm 1 Proposed model

Algorithm 1 combines the predictions from multiple machine learning models (Random Forest (RF), XGBoost, and Histogram-Based Gradient Boosting (HBGB)) using an ensemble averaging approach. Key steps include:

1. Training of multiple models.
2. Extracting the predicted probabilities from each model for every class.
3. Average probabilities for each class across all models.
4. The class with the highest average probability was selected for final prediction.
5. Finally, the optimal model was identified based on the performance metrics.

where n is the number of samples, d is the number of features, and t is the number of trees.

Table 6 shows the complex analysis of the random forest and XGBoost Models. The algorithm balances the predictions from RF, XGBoost, and HBGB to optimize the final performance. Although RF is computationally simpler, XGBoost offers superior accuracy for certain tasks at the expense of higher complexity. The ensemble approach ensures that the strengths of both models are utilized, and the optimal complexity depends on the performance-complexity trade-off demonstrated by the dataset.

**Table 6:** Complexity analysis of model.

Model	Time complexity	Space complexity	Training time (s)	Memory usage (Bytes)
Random forest	$O(n.d.t)$	$O(n.d)$	2	4848
XGBoost	$O(n.d.t)$	$O(n.d)$	2	96

The WVE classifier is an ensemble learning technique that combines the predictions of several base models. Each base model was assigned a weight based on its performance on the validation set. During prediction, the weighted votes from all base models were combined, and the class with the highest weighted vote was selected. The WVE can improve generalization, reduce overfitting, and increase robustness compared with individual models. We chose to use [Random Forest, eXtreme gradient boosting, and histogram-based gradient boosting] for WVE, as they have been shown to be effective in similar tasks.

A comparison of the accuracies of the proposed WVE classifier model and other models is presented in Table 7. The proposed method showed a significant improvement in accuracy compared with previous studies that used machine learning techniques for stroke detection.

**Table 7:** Performance comparison between proposed method and previous study.

Model	Accuracy	Study
Logistic regression	78.40%	^39^
Support vector machines	78.40%	^40^
Naïve bayes	77.40%	^41^
K-Nearest neighbor	91.72%	^42^
RF + XGBoost + HGB	92.31%	Current study

## Conclusion and future work

Preventive detection is essential for reducing brain damage and improving patient prognosis after stroke. Machine learning can play a key role in early stroke detection by analyzing medical data. In this study, a stroke recognition approach based on the WVE classifier was presented. The proposed model is a composite machine learning method. The integration of the results of several different classifiers, including Random Forest, eXtreme Gradient Boosting, and histogram-based gradient boosting, produces a definitive prediction. These probabilities, which all classifiers deliver as a valuation of the chance that they belong, are added to create the final forecast using a weighted average. Finally, our research provides a hybrid framework that integrates machine learning techniques. The improved weighted voting ensemble model classified brain stroke with high accuracy (92.31%). Our research provides a comprehensive understanding of patients’ overall health and potential risk factors.

### Limitation

One of the limitations of this study is the small size of our private dataset, which may affect the generalizability of the results. Furthermore, the complexity of combining multiple machine learning models in a weighted voting ensemble classifier to optimize accuracy is a challenge. Another drawback is the difficulty in understanding the hybrid RF + XGBoost + HGB models, which may make it difficult for healthcare providers to trust the model’s predictions. The stroke dataset used contained many samples labeled as"unknown,"and although data cleaning was performed, testing the models on different datasets might yield different results.

### Future work

Future research could develop an app for stroke diagnosis using CT scan image data to enhance patient outcomes and individualized treatment. The effectiveness of the framework should be assessed using larger datasets and for clinical utility in healthcare settings. The integration of advanced neuroimaging modalities and comprehensive clinical data could improve stroke localization and diagnostic accuracy. Privacy-preserving techniques, such as federated learning and centralized deep learning security exploration, can be adopted to maintain patient data confidentiality.

## Data Availability

The datasets generated and/or analysed during the current study are not publicly available due to that permission has not been granted by the hospital to share the dataset publicly, but are available from the corresponding author on reasonable request.
